# The relationship of general, physical, and psychological violence with depressive symptoms and cognition in elders (cross-sectional study)

**DOI:** 10.1590/0034-7167-2022-0375

**Published:** 2023-08-07

**Authors:** Rafael da Costa Santos, Gleicy Karine Nascimento De Araújo-Monteiro, Matheus Farias Raposo, Angela Maria Henao Castaño, Betânia Maria Pereira dos Santos, Rafaella Queiroga Souto

**Affiliations:** I Universidade Federal da Paraíba. João Pessoa, Paraíba, Brazil; II Universidad Nacional de Colombia. Bogotá, Colombia

**Keywords:** Violence, Depression, Cognition, Elderly, Forensic Nursing., Violencia, Depresión, Cognición, Anciano, Enfermería Forense., Violência, Depressão, Cognição, Idoso, Enfermagem Forense.

## Abstract

**Objectives:**

to analyze the relationship of general, physical, and psychological violence with depressive symptoms and cognition in the elderly.

**Methods:**

quantitative, cross-sectional study, carried out with 323 elders from the Brazilian northeast. Data collection used a sociodemographic instrument; the Conflict Tactics Scales Form R; the Geriatric Depression Scale; and the Mini-Mental State Examination. The analysis employed descriptive and inferential statistics.

**Results:**

as violence increases, so do the depressive symptoms; the opposite was true when comparing violence with cognitive involvement. There is a correlation between physical and psychological violence and depressive symptoms; more depressive elders are from 1.96 to 3.00 times more likely to be the victims of psychological and physical violence, respectively.

**Conclusions:**

general, physical, and psychological violence is associated with depressive symptoms; those with less cognitive alterations are more vulnerable to abuse. Elders with depressive symptoms are more likely to suffer psychological and physically violence.

## RESUMO

**Objetivos:**

analisar a relação da violência geral, física e psicológica com sintomas depressivos e estado cognitivo em pessoas idosas.

**Métodos:**

estudo quantitativo transversal, realizado com 323 idosos, do Nordeste brasileiro. Foram utilizados: instrumento sociodemográfico; *Conflict Tactics Scales Form R; Geriatric Depression Scale*; e Mini Exame do Estado Mental. Na análise, foi empregada estatística descritiva e inferencial.

**Resultados:**

à medida que aumenta a violência, aumentam os sintomas depressivos; o inverso ocorreu na avaliação da violência com o comprometimento cognitivo. Existe correlação entre violência física e psicológica e os sintomas depressivos; pessoas idosas mais depressivas têm 1,96 e 3,00 vezes mais probabilidade de sofrer violência psicológica e física, respectivamente.

**Conclusões:**

a violência geral, física e psicológica associa-se com os sintomas depressivos; e as pessoas com menos alterações cognitivas são mais vulneráveis ao abuso. Idosos com sintomas depressivos têm maior probabilidade de serem vítimas de violência psicológica e física.

## RESUMEN

**Objetivos:**

analizar la relación de la violencia general, física y psicológica con síntomas depresivos y estado cognitivo en personas ancianas.

**Métodos:**

estudio cuantitativo transversal, realizado con 323 ancianos, del Noreste brasileño. Fueron utilizados: instrumento sociodemográfico; *Conflict Tactics Scales Form R; Geriatric Depression Scale*; y Mini Examen del Estado Mental. En el análisis, fue empleado estadística descriptiva e ilativa.

**Resultados:**

a la medida que aumenta la violencia, aumentan los síntomas depresivos; el inverso ocurrió en la evaluación de la violencia con el comprometimiento cognitivo. Hay correlación entre violencia física y psicológica y los síntomas depresivos; personas ancianas más depresivas tienen 1,96 y 3,00 veces más probabilidad de sufrir violencia psicológica y física, respectivamente.

**Conclusiones:**

la violencia general, física y psicológica se relaciona con los síntomas depresivos; y las personas con menos alteraciones cognitivas son más vulnerables al abuso. Ancianos con síntomas depresivos tienen mayor probabilidad de ser violentados psicológica y físicamente.

## INTRODUCTION

Aging is an important factor to understand violence in the context of the elderly, since this stage of life is characterized by a reduction in one’s capacity to defend oneself and adapt. This also increases the state of organic vulnerability, leading to a loss of body homeostasis^(1)^. Therefore, violence is a global health issue; nonetheless, from a certain perspective, it is neglected^(2)^.

When a case of violence takes place within the spaces where the elder lives, the feeling of insecurity grows, with counterproductive impact on health, such as a considerable influence on the emergence of depressive symptoms^(3)^. This disorder is associated with other serious health issues, including death, lower quality of life, and disabilities^(4)^.

Mood changes due to depressive symptoms are elements from other complications in the health of the elder. In this setting, the cognitive decline stands out as a phenomenon that causes morbidity and mortality, worsens daily-life activities, social relations, and quality of life^(5)^.

Elderly with these health alterations (depressive symptoms and cognitive deficits) show higher levels of dependency. This lack of support demands considerably more from the caregiver, and can lead them to overload or trigger episodes of violence^(6-7)^, where physical violence is prevalent over psychological violence^(3)^.

This research is justified by the need to understand the relationship between the phenomenon mentioned above, in order to enable a change in the practice of assistance and provide integral care to elders who are vulnerable due to violence.

## OBJECTIVES

To analyze the relationship of general, physical, and psychological violence with depressive symptoms and cognition in the elderly.

## METHODS

### Ethical aspects

This study is part of the research “Use of Forensic Nursing in the care of hospitalized elders”, approved in the Research Ethics Committees (REC) of the participating institutions. All Directives from the National Council of Health for studies with human beings were respected, according to Resolution 466/12.

### Type of study

This is a quantitative, cross-sectional investigation, guided by the *Strengthening the Reporting of Observational Studies in Epidemiology* (STROBE). The study was carried out in the cities of João Pessoa and Campina Grande, in the state of Paraíba (PB), Brazil, from 2019 to 2020.

### Period and place of study

The research included elders attended in two teaching hospitals, one in each city mentioned above, regardless of the reason for their hospitalization. We chose these places as they are state references in the care of many medical specialties, especially in regard to the geriatric population.

### Population or sample; criteria of inclusion and exclusion

According with the frequency of monthly attention in the hospitals, the population of the study was formed by 1,259 elders: 774 from the hospital in João Pessoa, and 485 from the one in Campina Grande. To calculate the sample, we used a finite population formula for epidemiological studies, with a confidence index of 95% and an error of 5%, leading to a sample of 285 people: 193 from João Pessoa, and 120 from Campina Grande. We added 10% to this number to account for losses, which resulted in a final sample of 323 people.

We used a non-probabilistic sample by quota, proportionally distributing of the number of elders according with the sectors chosen in each unit where collection took place. The collection was carried out by convenience, due to the low turnover of elders in the services investigated.

The participants were aged 60 years or more and received hospital care in the chosen sectors. We excluded elders who were in terminal state (n=23), those whose communication capabilities were compromised (n=12), and those whose clinical characteristics prevented participation (n=11). The latter criteria were evaluated by the researcher who collected information or using information from the professionals in the service.

Data collection was carried out through a pilot test applied to 25 elders, aimed to calibrate any disagreements that might not have been identified during the organization of the survey. When there were no inconsistencies, the elders initially invited for the pilot test were also included in the number of the sentence.

All interviews were carried out by nursing undergraduates and MS and PhD students. All those responsible for data collection were trained to become familiar with the instrument used in the research. The interviews only took place after the participants received information about the objectives of the study; guidance regarding participation confidentiality and availability; and signed the Free and Informed Consent Form.

### Variables

The following instruments were used for data collection: a sociodemographic characterization form (variables - age, sex, reading and writing capabilities, marital state, living arrangements, work, and income); the Conflict Tactics Scales Form R (CTS - 1)^(8)^; the Geriatric Depression Scale (GDS)^(9)^; and the Mini-Mental State Examination (MMSE)^(10)^.

The CTS - 1 was elaborated in Canada. It is formed by 10 questions and subdivided in three groups based on the actions used to manage conflict situations, that is: arguments (items a c), verbal aggression (items d-f and h-j), and physical aggression (items k-s). Each question has three possible answers: Hasn’t happened; Has happened some times in the last 12 months; Has happened several times in the last 12 months. Regarding the reliability measures during the process of transcultural adaptation, the instrumented presented a Cronbach’s alpha near 0.70^(8)^.

The GDS is an instrument taht evaluates the presence or absence of depressive symptoms in the elderly population. This investigation adopted its version in 15 items, known as *Short Form* (GDS - 15). Its questions can be responded using two options: No (1) or Yes (2). The score is determined by adding 1 point to affirmative or negative responses, whenever they indicated the presence of depressive symptoms. The participant is classified in this instrument according to the score found: scores from 0 to 4 indicate no depression; from 5 to 10, depression; from 11 to 15, severe depression. The instrument was found to be relatively stable during the process of adaptation, as indicated by its results in Wilcoxon’s test (z = 1.60; p = 0.109), Spearman’s correlation (rho = 0.86; p < 0.001), and weighted Kappa (Kappa= 0.64)^(9)^. In regard to this data, the variable was transformed, and all participants whose score was ≥ 5 were classified as having “depressive symptoms”, with no indication of the intensity of said symptoms.

The MMSE was used to evaluate the cognitive state. The score of this scale varies from 0 to 30, and its classifications are directly dependent on the educational level of each interviewee, with a cutoff point of 13 for illiterate participants, 18 for those with a low educational level, and 26 for those with high educational level^(10)^. A previous study determined the reliability of the MMSE, finding a Kappa coefficient of 0.79, an interclass correlation coefficient of 0.80, and a Cronbach’s alpha of 0.71^(11)^.

### Result analysis

Data collected was double input by independent researchers in a statistical software; the divergence was reviewed and standardized by a data collection coordinator. Analyses were carried out using descriptive (relative and absolute frequencies) and inferential (Pearson’s chi-squared, Spearman’s correlation, and logistic regression) statistics. In all analyses, a significance level of 5% (p<0.05) was considered.

## RESULTS

Most participants were female elders (196; 60.7%); with a maximum age of 70 years old (170; 52.6%; who were literate (219; 67.8%); had a partner (167; 51.9%); lived with someone (288; 89.2%); and earned up to one minimum wage (187; 57.9%). Violence was present in 55.1% (n=178) of interviewees.

Table 1 shows the association between sociodemographic characteristics and violence. There was no statistically significant association between variables. The analysis of violence shows that its percentage was the same in both sexes, but predominates in 70-year-old or younger elders, who can read and write, have no partner, live alone, and earn more than one minimum wage.

**Table 1:** Association between violence and the sociodemographic characteristics of the elder participants of this research, João Pessoa and Campina Grande, Paraíba, Brazil, 2019-2020, n = 323

Variables	Violence		*p* value
	With violence	W/o violence	
Sex	n (%)	n (%)	
Female	108 (55.1)	88 (44.9)	0.545
Male	70 (55.1)	57 (44.9)	
Age			
≤ 70 years	99 (58.2)	71 (41.8)	0.263
> 70 years	79 (51.6)	74 (48.4)	
Knows how to read and write			
Yes	123 (56.2)	96 (43.8)	0.632
No	55 (52.9)	49 (47.1)	
Marital Status			
Has a partner^†^	90 (53.9)	77 (46.1)	0.654
Does not have a partner^‡^	88 (56.8)	67 (43.2)	
Living arrangements			
Lives alone	21 (60.0)	14 (40.0)	0.592
Lives with someone	157 (54.5)	131 (45.5)	
Monthly Income			
Up to one minimum wage	97 (51.9)	90 (48.1)	0.176
More than one minimum wage	81 (59.6)	55 (40.4)	

* *Person’s chi-squared test; †Married/Lives with someone; ‡Widow/divorced/never married.*

Table 2 shows the result of the correlation test between the total scores of the instruments GDS-15, MMSE, and CTS, also considering physical and psychological violence. It can be noted that, as general violence increases, depressive symptom scores also are raised. Nonetheless, in the evaluation of the MMSE, the result shows that violence is more present in elders with less cognitive impairments. Specifically in regard to physical and psychological violence, there is a correlation with depressive symptoms, and the elders who are more cognitively impaired are more vulnerable to situations of physical violence.

**Table 2:** Correlation of total CTS scores, and physical and psychological violence with the total GDS-15 and MMSE scores, João Pessoa and Campina Grande, Paraíba, Brazil, 2019-2020, n= 323

Variables	Correlation coefficient	*p* value
	Total CTS score^*^	
Total GDS-15 score^‡^	0.179	**0.001**
MMSE total score^§^	0.116	0.037
	Physical violence	
Total GDS-15 score^‡^	0.187	**0.001**
MMSE total score^§^	-0.065	0.241
	Psychological violence	
Total GDS-15 score^‡^	0.183	**0.001**
MMSE total score^§^	0.105	0.059

* *Conflict Tactic Scale; †Spearman’s correlation test; ‡Geriatric Depression Scale; §Mini-Mental State Examination.*

There was an association between types of violence (physical and psychological) as evaluated by the CTS, depressive symptoms, and cognitive deficits, as Table 3 shows. There was a statistically significant association between physical and psychological violence and the variable “depressive symptoms”, according to which violence was more prevalent in those “with depressive symptoms”. Regarding the variable “cognitive deficit”, there was no association, and physical violence predominated in those who presented cognitive deficits; psychological violence, on the other hand, predominated in elders with no cognitive commitments.

**Table 3:** Association of general, physical, and psychological violence with depressive symptoms and cognitive deficits, João Pessoa and Campina Grande, Paraíba, Brazil, 2019-2020, n = 323

Variables	With violence	W/o violence	*p* value
	n (%)	n (%)	
General violence			
Depressive symptoms			0.004
With depressive symptoms	97 (63.8)	55 (36.2)	
W/o depressive symptoms	81 (47.4)	90 (52.6)	
Cognitive deficit			0.902
With cognitive deficits	50 (54.3)	42 (45.7)	
W/o cognitive deficits	128 (55.4)	103 (44.6)	
Physical violence			
Depressive symptoms			0.003
With depressive symptoms	26 (17.1)	126 (82.9)	
W/o depressive symptoms	11 (6.4)	160 (93.6)	
Cognitive deficit			0.699
With cognitive deficits	12 (13.0)	80 (87.0)	
W/o cognitive deficits	25 (10.8)	206 (89.2)	
Psychological violence			
Depressive symptoms			0.004
With depressive symptoms	97 (63.8)	55 (36.2)	
W/o depressive symptoms	81 (47.4)	90 (52.6)	
Cognitive deficit			0.902
With cognitive deficits	50 (54.3)	42 (42.7)	
W/o cognitive deficits	128 (55.4)	103 (44.6)	

* *Pearson’s chi-squared test.*

Table 4 shows the logistic regression model for physical and psychological violence. In it, the variable “depressive symptoms” was inserted due to the fact it presented a *p*-value < 0.2 in the bivariate analysis.

**Table 4:** Variable associated with psychological and physical violence after an adjusted logistic regression, João Pessoa and Campina Grande, Paraíba, Brazil, 2019-2020

Variables	OR†	CI‡	*p* value
Psychological violence			
Depressive symptoms			
With symptoms	1.96	1.25-3.06	0.003
W/o symptoms	1.00	-	-
Physical violence			
Depressive symptoms			
With symptoms	3.00	1.42-6.30	0.004
W/o symptoms	1.00	-	-

*†Odds Ratio; ‡Confidence interval.*

The data allow us to point out that elders with depressive symptoms are 1.96 and 3.00 times more likely to be victims of psychological and physical violence, respectively.

## DISCUSSION

As we analyze the sociodemographic variables, the percentage of aggression in individuals was the same for both sexes, although literature shows elderly women as more likely victims of abuse^(7)^. Being younger than 71 was also associated with a higher prevalence of violence; a systematic review study showed that age is associated with violence, but the documents found are not in agreement when it comes to age group: from the four documents, two reported that being a younger elder (< 70 years) and was a risk factor, while the other two mentioned it as a protective factor^(12)^. This disagreement may be associated with the specific characteristics of each population, since these investigations were carried out in different countries.

Literate elders also were more likely to be the victims of violence. This evidence is similar to the findings of a study carried out using data from the Brazilian Institute of Geography and Statistics (IBGE), which shows an association between a higher educational level and violence^(3)^. The violence was also more prevalent in elders who had no partner or lived alone. A Saudi study reiterates this finding, reporting that single and widow/widower elders are, respectively, 6.10 and 2.96 times more likely to be abused^(13)^; for elders who live alone, the likelihood is 10.25 times greater^(12)^.

Studies have shown that income is strongly associated to abuse, with low income and financial dependency as risk factors^(12,14-15)^. This disagrees with our results, as we found that elders with more than one minimum wage were more often the victims of violence. An explanation to this disagreement could be in the increased (albeit still insufficient) economic power of the Brazilian population, and on the increased dependency of other relatives on the income of the family elderly, which makes violence more likely^(16-17)^.

Those who reported being the victims of violence also showed a high percentage of depressive symptoms. This disorder can trigger several issues associated with the health of the elderly. Other studies have showed that depressive symptoms are directly associated with chronic diseases, social distancing, emotional disorders caused by social factors, homeostasis changes, and other losses^(7,18)^ - problems that can be made worse by violence.

The same is true when we analyze the type of violence: regardless of whether the violence was physical or psychological, depressive symptoms will be related. Psychological abuse is the most associated with factors that intervene in the quality of life of the elder, such as depression and anxiety^(19)^.

Physical abuse is quite frequent in this population, being the most prevalent characteristic in the elderly, with a history of repeated offenses. Furthermore, several psychiatric conditions can be noticed, such as: depression, psychosis, and anxiety^(20)^. This study also shows that, as physical violence cases increase, depressive symptoms also increase.

Regarding cognitive deficits, they are a very common phenomenon in the aging process. When this is evaluated together with violence, we can notice that elders with cognitive impairments are more often the victims of violence^(21)^. Nonetheless, our results show that those with less cognitive impairments were the most likely to suffer general violence. Only in regard to physical violence, specifically, elders with more cognitive impairment were more often the victims.

Some studies, including a systematic review, also reiterated the information that elders with suggestive cognitive deficit alterations are more likely to undergo some type of violence^(12,15,22)^. This disagreement with our research can be due to the population studied, denoting the need for further research that detail this phenomenon, presenting an overview of this relationship between violence and cognitive impairments.

There is a strong connection between the factors studied here, which can generate a perpetuation of this cycle of violence. This is because literature - which was corroborated by the data found in this investigation, which associates violence and depressive symptoms - describes a path where the chances for a person with psychiatric disorders to be the victim of violence is 7.1 times higher than that of an individual with no mental disorders^(23)^. In this investigation, we can see that, regarding the association of depressive symptoms and psychological and, especially, physical violence, depressive elders were also, respectively, 1.96 and 3.00 more likely to be the victims of each type of violence.

Future investigations must be conducted to evaluate further the phenomena presented here. The relationship between these elements is still not well defined, and, to make matters worse, they are in a feedback cycle, meaning that depressive symptoms and forms of violence seem increase the impact of one another^(24)^.

### Study limitations

The limitations of this research were as follows: the data was self-reported by the elders, in some cases with cognitive disorders, and, for them, it may be taboo to talk about the violence to which they were submitted; there was a lack of studies that evaluate violence and the other variables presented here, especially in the case of physical and psychological violence; we did not control for confounding factors; the sample was by convenience, due to the situation of the hospitals, which had a low turnover of hospitalized elders.

### Contributions for the field

The study enables evaluating violence not only in general, but also its physical and psychological manifestations. It allows nursing workers to understand how this phenomenon interacts with cognition and with the depressive symptoms of the elders, and can generate factors that aid in the elaboration of a plan of care individualized to the elders who are victims of violence.

## CONCLUSIONS

Considering the elders evaluated in this research, we can say that those with the most depressive symptoms were more likely to be the victims of violence, with these symptoms are associated with physical and psychological violence. Regarding cognitive impairment and violence, elders with less alterations were more vulnerable to abuse, and those with more depressive symptoms were more likely to be victims of psychological and physical violence.
